# Taste Receptor Activation in Tracheal Brush Cells by Denatonium Modulates ENaC Channels via Ca^2+^, cAMP and ACh

**DOI:** 10.3390/cells11152411

**Published:** 2022-08-04

**Authors:** Monika I. Hollenhorst, Praveen Kumar, Maxim Zimmer, Alaa Salah, Stephan Maxeiner, Mohamed Ibrahem Elhawy, Saskia B. Evers, Veit Flockerzi, Thomas Gudermann, Vladimir Chubanov, Ulrich Boehm, Gabriela Krasteva-Christ

**Affiliations:** 1 Institute of Anatomy and Cell Biology, Saarland University, 66421 Homburg, Germany; 2 Institute for Experimental and Clinical Pharmacology and Toxicology, Centre for Molecular Signalling, Saarland University, 66421 Homburg, Germany; 3 Walter-Straub-Institute for Pharmacology and Toxicology, Ludwig-Maximilians-University and German Centre for Lung Research (DZL), 80366 Munich, Germany; 4 Experimental Pharmacology, Centre for Molecular Signalling, School of Medicine, Saarland University, 66421 Homburg, Germany

**Keywords:** ion transport, airway epithelium, Ussing chamber, mucociliary clearance, brush cell, ENaC, ACh, Trpm5, CFTR

## Abstract

Mucociliary clearance is a primary defence mechanism of the airways consisting of two components, ciliary beating and transepithelial ion transport (I_SC_). Specialised chemosensory cholinergic epithelial cells, named brush cells (BC), are involved in regulating various physiological and immunological processes. However, it remains unclear if BC influence I_SC_. In murine tracheae, denatonium, a taste receptor agonist, reduced basal I_SC_ in a concentration-dependent manner (EC_50_ 397 µM). The inhibition of bitter taste signalling components with gallein (G_βγ_ subunits), U73122 (phospholipase C), 2-APB (IP3-receptors) or with TPPO (Trpm5, transient receptor potential-melastatin 5 channel) reduced the denatonium effect. Supportively, the I_SC_ was also diminished in Trpm5^−/−^ mice. Mecamylamine (nicotinic acetylcholine receptor, nAChR, inhibitor) and amiloride (epithelial sodium channel, ENaC, antagonist) decreased the denatonium effect. Additionally, the inhibition of G_α_ subunits (pertussis toxin) reduced the denatonium effect, while an inhibition of phosphodiesterase (IBMX) increased and of adenylate cyclase (forskolin) reversed the denatonium effect. The cystic fibrosis transmembrane conductance regulator (CFTR) inhibitor CFTR_inh172_ and the KCNQ1 potassium channel antagonist chromanol 293B both reduced the denatonium effect. Thus, denatonium reduces I_SC_ via the canonical bitter taste signalling cascade leading to the Trpm5-dependent nAChR-mediated inhibition of ENaC as well as G_α_ signalling leading to a reduction in cAMP-dependent I_SC_. Therefore, BC activation contributes to the regulation of fluid homeostasis.

## 1. Introduction

Mucociliary clearance (MCC) is part of the innate immune processes of the airways, in which a thin layer of mucus secreted by goblet cells and submucosal glands traps inhaled pathogens and particles. In the healthy airway epithelium, MCC depends on ciliary beat and transepithelial ion transport. The regulation of transepithelial ion transport, which results in passive water movement across the epithelial layer, is essential for providing the optimal milieu for effective ciliary beat [1]. In the airways, the height of the periciliary liquid (PCL) is mainly regulated by sodium and chloride transport [2]. Sodium absorption is mainly mediated by the epithelial sodium channel (ENaC), and chloride secretion by the cystic fibrosis transmembrane conductance regulator (CFTR) and calcium-dependent chloride channels (CaCC), especially transmembrane protein 16A (TMEM16A) [3]. The maintenance of apical chloride secretion is dependent on the basolateral Na-K-2Cl co-transporter as well as on basolateral potassium secretion via the potassium voltage-gated channel subfamily Q member 1 (KCNQ1) channel [3,4,5]. Several diseases, such as primary ciliary dyskinesia, asthma, cystic fibrosis (CF), and chronic obstructive pulmonary disease (COPD), are accompanied by impaired MCC that leads to disease worsening and exacerbation [6,7,8].

Recently, cholinergic chemosensory cells in the airways consisting of brush cells (BC) in the lower and solitary chemosensory cells in the upper respiratory tract were found to regulate the MCC [9,10]. Moreover, these cells detect bitter substances and bacterial quorum-sensing molecules displaying bitter characteristics using bitter taste signalling involving the transient receptor potential melastatin 5 (Trpm5) channel [11,12,13,14]. They further seem to be important for provoking immune responses for pathogen elimination [11,12,13,14]. In the trachea, the stimulation of BC with Tas2R agonists, e.g., denatonium and the formylated peptide f-MKKFRW, triggers a PLC_β2_-mediated release of Ca^2+^ from inositol 1,4,5-trisphosphate (IP3)-sensitive Ca^2+^ stores, resulting in Trpm5 channel activation and acetylcholine (ACh) release from BC [9,10,15]. The released ACh then stimulates the cilia-mediated part of the MCC via muscarinic (m)3 and nicotinic (n) ACh receptors (AChR) in ciliated cells [9,10,16]. Similarly, in the upper respiratory tract, solitary chemosensory cells are also able to modulate the cilia-dependent part of MCC [11,12]. Additionally, ACh acts in an autocrine manner on BC. While the m1- and m3AChR are activating, m2AChR is inhibitory [10]. Recently, we found that also nAChR can regulate the transepithelial ion transport [17,18]. Additionally, a recent study points towards an influence of denatonium on transepithelial ion transport in human upper airway cultures [19]. However, it remains unclear whether BC-derived ACh is, indeed, involved in the regulation of transepithelial ion transport.

In this study we evaluate the role of BC on transepithelial ion transport processes as a part of MCC. We focused in particular on elucidating the intracellular signalling cascade involved in the ion transport changes observed upon the application of the BC agonist denatonium and the participating ion channels.

## 2. Materials and Methods

### 2.1. Animals

All procedures were carried out in accordance with the German animal welfare law. The resiniferatoxin protocol was approved by the Animal Welfare Committee of Saarland (protocol number: 25-2021). Male and female 10–16 weeks aged wildtype (wt, C57Bl/6J, RRID:IMSR_JAX:000664), Trpm5^−/−^ [20], and Trpm5-tauGFP [21] mice were used in this study. The Trpm5-tauGFP mice were crossbred from Rosa26-tauGFP mice [22] and Trpm5-IREScre mice [23]. Once the mice reached the appropriate age, they were randomly chosen for experiments. Wt and the Trpm5^−/−^ mice were raised in IVC cage systems in the Research Animal Facility of the Institute of Experimental Surgery of Saarland University. The Trpm5-tauGFP mice were bred and kept under SPF conditions in the Research Animal Facility of the Institute of Experimental and Clinical Pharmacology of Saarland University. All mice were housed in groups of four to five mice per cage with a standard 12 h light:12 h dark cycle and provided food and water ad libitum.

### 2.2. Reagents

Reagents were purchased as follows: amiloride, ACh, 2-APB, ATP, atropine, BaCl_2_, calcium D-gluconate, denatonium, D-glucose, DMSO, glibenclamide, 3-isobutyl-1-methylxanthine, mannitol, mecamylamine, NaHCO_3_, NaOH, pluronic, forskolin, pyruvic acid, bumetanide, CFTR_inh172_, and TPPO from Sigma-Aldrich (Taufkirchen, Germany); gallein, NPPB, chromanol 293B, and pertussis toxin from Tocris Bioscience (Abingdon, UK); carbenoxolone disodium salt from Abcam (Cambridge, UK); KH_2_PO_4_, MgCl_2_, and KCl from MERCK (Darmstadt, Germany); U-73122 from Calbiochem (MERCK); MK-886 from Millipore (MERCK); NaCl and CaCl_2_ from Grüssing GmbH (Filsum, Germany); HEPES from Carl Roth (Karlsruhe, Germany); thapsigargin from Cayman Chemicals (Ann Arbor, MI, USA); EDTA from VWR (Darmstadt, Germany); DMEM, DPBS, FBS, antibiotic antimycotic, optiMEM, and sodium pyruvate from Gibco (Thermo Fisher Scientific, Waltham, MA, USA); lipofectamine 2000 and fura-2 from Invitrogen (Thermo Fisher Scientific); resiniferatoxin from AdipoGen (Biomol, Hamburg, Germany). 

### 2.3. Ussing Chamber Experiments

Ussing chamber experiments were performed as described previously [18]. Briefly, mice were killed by inhalation of an overdose of the narcotic isoflurane (Piramal Critical Care Deutschland GmbH, Hallbergmoos, Germany) followed by aortic exsanguination. The trachea was explanted, cut longitudinally and mounted onto a modified Ussing chamber. Then, it was perfused with a buffer solution containing (in mM) 145 NaCl, 1.3 Ca^2+^ gluconate, 1 MgCl_2_, 5 HEPES, 5 D-glucose and 2 KH_2_PO_4_ warmed to 37 °C with a pH of 7.4. The tracheae were perfused for 10 to 15 min with the buffer for equilibration of the baseline before an apical application of denatonium for 5 min. Denatonium was washed for 10 to 15 min. Tracheae were perfused with vehicle or different antagonists for 10 to 15 min according to the protocol (apical: gallein, U73122, 2-APB, thapsigargin, carbenoxolone, TPPO, MK-886, pertussis toxin, IBMX, amilorid, NPPB, CFTR_inh172_ or BaCl_2_; basolateral: bumetanide, BaCl_2_, chromanol 293B or TPPO; apical and basolateral: mecamylamine or atropine). Then, denatonium was applied in the presence of the antagonists apically for 5 min. The tracheae were washed again for 10 min. The viability of the tissue was controlled at the end of each experiment by ATP (apical) stimulation for 5 min. For measuring the short circuit current (I_SC_), we used a voltage clamp amplifier (KU Leuven, Belgium) connected via the analogue-to-digital converter PowerLab version 4/35 (ADInstruments, Spechbach, Germany) to a MS-DOS computer with LabChart version 8 (ADInstruments). For all experiments the mice were randomly chosen and the experiments with wt and Trpm5^−/−^ mice were performed blindly. 

### 2.4. Cloning

Expression vectors with the coding sequence of mouse *Tas2r105* and *Tas2r108* genes were cloned using commercial DNA assembly strategies, according to manufacturer’s recommendation (NEBuilder HiFi, New England Biolabs GmbH, Frankfurt, Germany). The generation of PCR products followed standard procedures using PrimeStar HS DNA Polymerase (TaKaRa Inc., Otsu, Japan). Oligonucleotides were designed based on taste receptor sequences deposited to and annotated in the NCBI database (*Tas2r105* Gene ID 57252, and *Tas2r108* Gene ID 57253; www.ncbi.nlm.nih.gov/gene, accessed on 4 June 2021). Oligo combinations MX18750/51 and MX18752/53 were used to amplify the *Tas2r105* and *Tas2r108* sequences, respectively. The taste receptor sequences are localized on a single exon; hence, amplification was performed using C57BL/6 genomic DNA as a template source. Oligo combination MX18748/49 was used to amplify the vector backbone from a pcDNA3.1 template preparing the vector to home both taste receptor sequences. The list of oligonucleotide sequences can be found in the Supporting Material (Table S1).

### 2.5. Calcium Imaging Experiments

HEK-293 cells were transfected with Tas2r105- or Tas2r108-coding plasmids using Lipofectamine 2000, according to the manufacturer’s protocol. DNA and Lipofectamine were diluted in Opti-MEM and incubated with the cells overnight. Calcium imaging experiments were performed as described previously [18]. Briefly, coverslips seeded with transfected and non-transfected HEK293 cells were washed three times with Tyrode III buffer containing (in mM) 130 NaCl, 10 HEPES, 10 glucose, 8 CaCl_2_, 5 KCl, 10 sodium pyruvate, 5 NaHCO_3_, and 1 MgCl_2_. Then, cells were loaded with Fura-2-AM (5 µM, Invitrogen) and 0.04% pluronic (Sigma-Aldrich) for 30 min at 37 °C in Tyrode III buffer. Changes in [Ca^2+^]_i_ levels to denatonium application were studied using an Eclipse FN1 microscope (Nikon Instruments, Amsterdam, The Netherlands). Cells were excited alternately at 340 and 380 nm using Lambda DG-4 high-speed wavelength switcher equipped with a xenon arc lamp (Sutter Instruments, Novato, CA, USA), and emission was detected at 510 nm using an Orca Flash 4.0 camera (Hamamatsu, Herrsching, Germany). Acquired values were processed using the NIS-Elements software (Nikon Instruments, Amsterdam, The Netherlands).

### 2.6. Ablation of Sensory Nerves with Resiniferatoxin

Trpm5-tauGFP mice were injected s.c. with increasing concentrations of resiniferatoxin (RTX, 30, 70, and 100 µg/kg) on three consecutive days under isoflurane narcosis (starting initially with 3% with a flow rate of 1 L/min followed by 2% with the same flow rate for 3 min). The mice were given Tramal (1 mg/mL, Grünenthal, Aachen, Germany) in the drinking water one day prior to the first RTX injection until the day of the last injection of RTX. One week after the last injection, mice were sacrificed with an overdose of isoflurane and the tracheae were used for Ussing chamber studies. To confirm successful nerve ablation, subsequently to the Ussing chamber experiments, the tracheae were fixed in Zamboni fixative consisting of 2% paraformaldehyde (PFA) and 15% saturated picric acid in 0.1 M phosphate buffer (pH 7.4), followed by three days of washing in 0.1 M phosphate buffer (pH 7.2) before being subjected to whole-mount staining. 

For the further validation of ablation of sensory nerves, the immunohistochemistry of dorsal root ganglia (DRG) sections was performed as described previously [14]. Briefly, cryo-sections of DRG were incubated for 2 h with a blocking solution containing 2% horse serum and 0.1% TritonX-100 in PBS. Sections were incubated over night at room temperature with primary antisera against Trpv1 raised in rabbit (dilution 1:1600, Alomone labs, Jerusalem, Israel). After washing in PBS, tissue sections were incubated for 1 h with anti-rabbit secondary antisera (donkey anti-rabbit IgG antibody, Cy3-conjugated, dilution 1:500, Merck Millipore, Darmstadt, Germany) and finally with DAPI (Carl Roth, Karlsruhe, Germany) for 10 min. After a washing step, the slides were coverslipped with Mowiol 4-88 (Carl Roth) and evaluated on an Axio Imager M2 (Zeiss, Oberkochen, Germany) fluorescence microscope using an Axiocam 512 colour (Zeiss) camera and the Zen 2.3 software (Zeiss).

### 2.7. Whole-Mount Staining

Trpm5-tauGFP mice were used to visualize tracheal BC by their green fluorescence. Control Trpm5-tauGFP and RTX-treated Trpm5-tauGFP mice were sacrificed by inhalation of an isoflurane overdose followed by aortic exsanguination. The trachea was dissected, opened longitudinally, divided into two pieces, and fixed in Zamboni fixative as described above. The tissue was permeabilised with 0.3% Triton X-100 (Carl Roth) in PBS for 2 h and incubated with 1% BSA and 4% horse serum in PBS for 2 h at room temperature. Next, the primary antisera against CGRP raised in goat (dilution 1:1000, Acris, Origene, Herford, Germany), against PGP9.5 raised in rabbit (dilution 1:800, abcam, Berlin, Germany) and GFP raised in chicken (dilution 1:500, Novusbio, Abingdon, UK) were applied overnight at room temperature. The tissue was washed 5 times with PBS for 30 min each and then incubated with Cy3-conjugated donkey anti-goat IgG (dilution 1:200, Merck Millipore, Darmstadt, Germany), Cy5-conjugated donkey anti-rabbit IgG (dilution 1:200, Jackson ImmunoResearch, Ely, UK) and FITC-conjugated donkey anti-chicken IgG (dilution 1:200, Jackson ImmunoResearch) overnight at room temperature. Finally, the tracheal pieces were incubated with DAPI (0.2 µg/mL) for 30 min followed by 5 washing steps in PBS for 30 min each prior to mounting on glass slides with Mowiol 4-88 (Carl Roth, Karlsruhe, Germany). Image acquisition was performed using a Leica TCS SP5 Confocal Laser Scanning Microscope (Leica Microsystems, Wetzlar, Germany) and the ImageJ (NIH, Bethesda, MD, USA) software was used for analysis.

### 2.8. Experimental Design and Statistical Analysis

All values were calculated for 1 cm^2^ tissue area in the Ussing chamber experiments. At least 5–6 biological replicates for all experiments were measured. Data are depicted as mean ± standard error of the mean (*n* = number of measured tracheae or cells). The Kolmogorov–Smirnov test was used to study the normal distribution of the data. Then, the paired or unpaired Student’s *t*-test was applied to investigate statistical differences. *p* < 0.05 was considered statistically significant.

## 3. Results

### 3.1. Denatonium Modulates Transepithelial Ion Transport in Wildtype Mice

**Figure 1:**
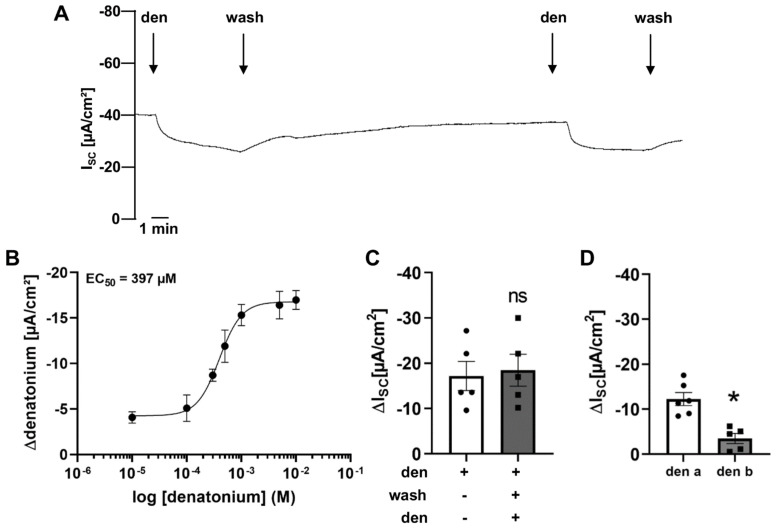
Effect of the bitter taste receptor agonist denatonium on transepithelial ion transport of mouse tracheal epithelium. (**A**) Apical application of denatonium (1 mM) resulted in a decrease in the net short circuit current (I_SC_). Representative current trace. (**B**) The denatonium-induced effect was dose-dependent with an EC_50_ of 397 µM in wildtype mouse tracheae (*n* = 5 for each concentration). (**C**) Repeated application of denatonium (1 mM, apical) on the same trachea showed similar denatonium-modulated current changes (ΔI_SC_) upon the second application (*n* = 5, ns: not significant). (**D**) The current changes (ΔI_SC_) upon basolateral application of denatonium (1 mM, *n* = 5) were smaller than upon apical application (* *p* < 0.05).

Initially, we studied the basic effect of denatonium on transepithelial ion current. The apical application of 1 mM denatonium decreased the net I_SC_ by −17.20 ± 3.21 µA/cm^2^ compared to the baseline (Figure 1A). The denatonium-induced effect was concentration-dependent with an EC_50_ of 397 μM (Figure 1B). The response to repetitive apical application of 1 mM denatonium (−18.45 ± 3.51 µA/cm^2^ response for the second application) did not differ compared to the first application (Figure 1C). The basolateral application of 1 mM denatonium reduced the net I_SC_ as well. Nevertheless, this response amounted less than 30% in comparison to the effect of denatonium when it was applied apically (Figure 1D).

### 3.2. Denatonium Acts via the Canonical Bitter Taste Signalling Cascade

**Figure 2:**
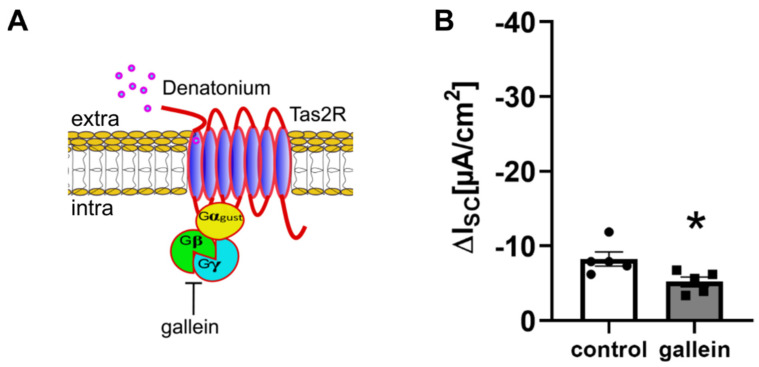
Denatonium acts on G protein-coupled taste receptors. (**A**) Schematic drawing of the action of the G_βγ_ inhibitor gallein. (**B**) The G_βγ_ subunit antagonist gallein (50 µM, *n* = 5, apical) reduced the denatonium-induced current (∆I_SC_, *n* = 5, * *p* < 0.05).

**Figure 3:**
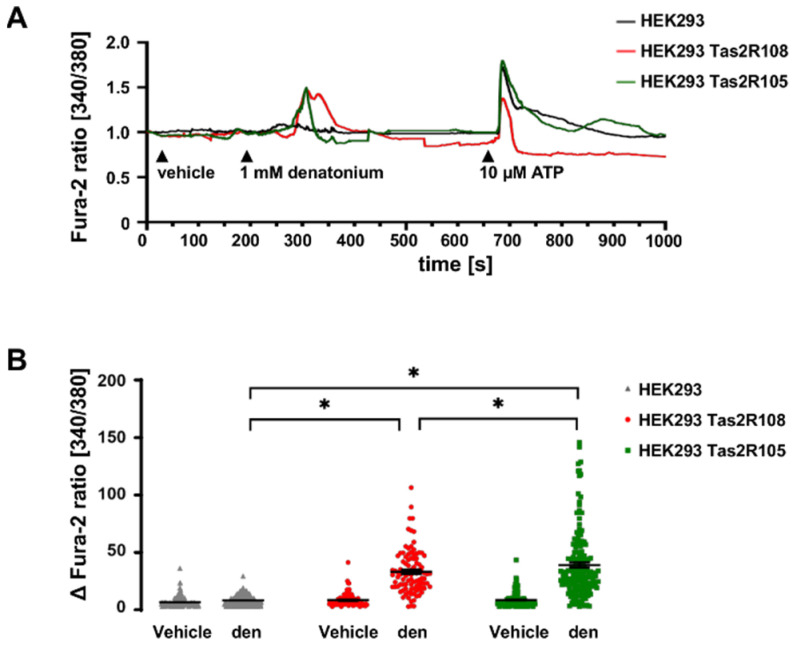
Denatonium activates Tas2R108 and Tas2R105. (**A**) Application of denatonium (1 mM) increased [Ca^2+^]_i_ in HEK293 cells transfected with Tas2R108 (red) or Tas2R105 (green) but not in untransfected HEK293 cells. The ATP (10 µM) control stimulus increased [Ca^2+^]_i_ in all cell lines. Representative curves for the Ca^2+^ response. (**B**) The denatonium-induced [Ca^2+^]_i_ was increased in the HEK293 cells transfected with Tas2R108 (*n* = 99) or Tas2R105 (*n* = 160) compared to untransfected HEK293 cells (*n* = 227) and the [Ca^2+^]_i_ response to denatonium was higher in HEK293 Tas2R105 cells than in HEK293 Tas2R108 cells (* *p* < 0.05).

BC express components of the canonical bitter taste signalling cascade as revealed previously by single-cell RNAseq [10,24,25,26]. Here, we investigated the involvement of this cascade in the denatonium-induced ion transport changes. The administration of 50 µM gallein, which blocks beta and gamma subunits of G proteins [27], reduced the effect of denatonium by 40% (Figure 2A,B), indicating that denatonium acts via G protein-coupled receptors. Tas2R108 is the main Tas2R in tracheal BC as revealed previously by single-cell RNAseq [10]. The application of denatonium (1 mM) increased [Ca^2+^]_i_ in HEK293 cells expressing Tas2R108 or Tas2R105, while [Ca^2+^]_i_ levels in untransfected cells remained unaltered (Figure 3A,B). All cells reacted to the stimulation with the control stimulus ATP (10 µM) (Figure 3A).

**Figure 4:**
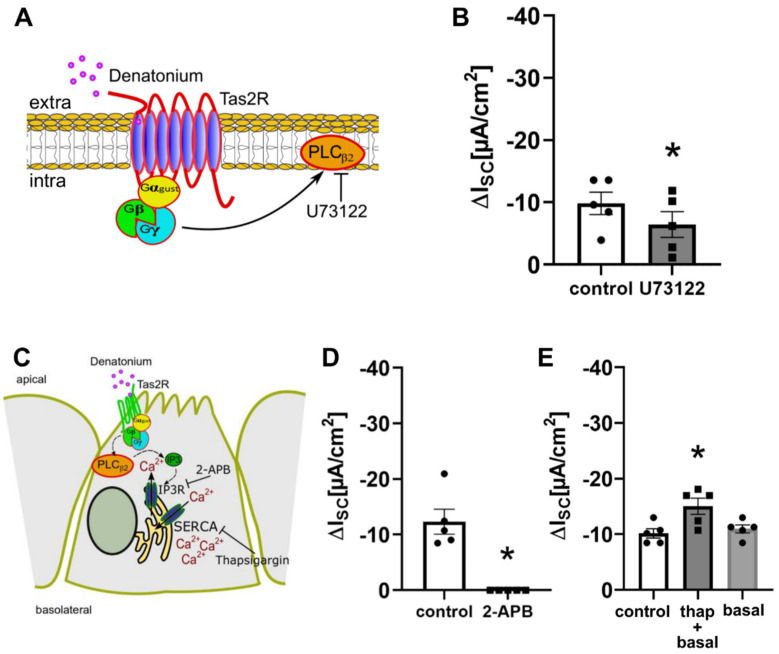
Involvement of components of the canonical bitter taste signalling cascade in the denatonium-induced effect. (**A**) Schematic drawing of the action of the PLC_β2_ inhibitor U-73122. (**B**) In the presence of the PLC_β2_ inhibitor U-73122 (10 µM, apical), the denatonium-induced current was reduced compared to control conditions (∆I_SC_, *n* = 5, * *p* < 0.05). (**C**) Schematic drawing of the influence of the 1,4,5-trisphosphate (IP3) receptor inhibitor 2-APB, the gap junction inhibitor carbenoxolone and the SERCA inhibitor thapsigargin. (**D**) The IP3 receptor inhibitor 2-aminoethoxydiphenyl borate (2-APB, 100 µM, apical) reduced the denatonium-induced effect (ΔI_SC_, *n* = 5, * *p* < 0.05). (**E**) Depletion of endoplasmic reticulum Ca^2+^ stores with 1µM thapsigargin (thap, apical) in the presence of extracellular Ca^2+^ did not impact the denatonium-induced effect (ΔI_SC_, *n* = 5, * *p* < 0.05). Control: denatonium-effect without thapsigargin, thap + basal: denatonium-effect in the presence of thapsigargin, basal: denatonium-effect in the presence of thapsigargin without the thapsigargin-induced increase of basal I_SC_.

To elucidate whether phospholipase C is involved in the denatonium-induced ion current changes, we performed experiments with the PLC inhibitor U-73122 (10 µM, apical, Figure 4A,B). In the presence of the inhibitor, the denatonium-induced effect was reduced compared to the control conditions, showing the involvement of PLC_β2_.

The activation of PLC_β2_ leads to the cleavage of phosphatidylinositol 4,5-bisphosphate into diacylglycerol and inositol 1,4,5-trisphosphate (IP3). IP3 then binds to its receptors in the endoplasmic reticulum, which leads to a release of Ca^2+^. To elucidate the role of Ca^2+^ in the denatonium-induced current changes, we performed experiments with the IP3 receptor inhibitor 2-aminoethyl-diphenyl-borinate (2-APB, 100 µM, apical) [28], which strongly reduced the denatonium-induced effect (Figure 4C,D), indicating a dependency of the denatonium-induced current changes on the release of Ca^2+^ from intracellular stores. However, 2-APB can also inhibit the store-operated plasma membrane Ca^2+^ channels Orai and TRPC at the concentration used here [29,30], strongly suggesting also an involvement of extracellular Ca^2+^ in the denatonium-induced effect. Indeed, Orai channels are expressed in BC as well as in ciliated cells according to our previous sequencing data [10]. Thapsigargin (1 µM), an inhibitor of the SERCA Ca^2+^ ATPase in the endoplasmic reticulum (ER) [31], which blocks the Ca^2+^ reuptake in the ER but also activates store-operated Ca^2+^ entry (SOCE) involving the ER Ca^2+^ sensor STIM1 and Orai1 [32,33], led to an elevation of I_SC_ approximately by 20% when it was perfused apically for 10 min. Interestingly, the denatonium-induced effect after thapsigargin was increased to the same extend. It is tempting to speculate that the presence of a denatonium-induced effect after ER depletion is most probably due to the presence of Ca^2+^ in our perfusion buffer and the refilling of ER through SOCE (Figure 4C,E) [34] or to an incomplete emptying of ER Ca^2+^ stores.

**Figure 5:**
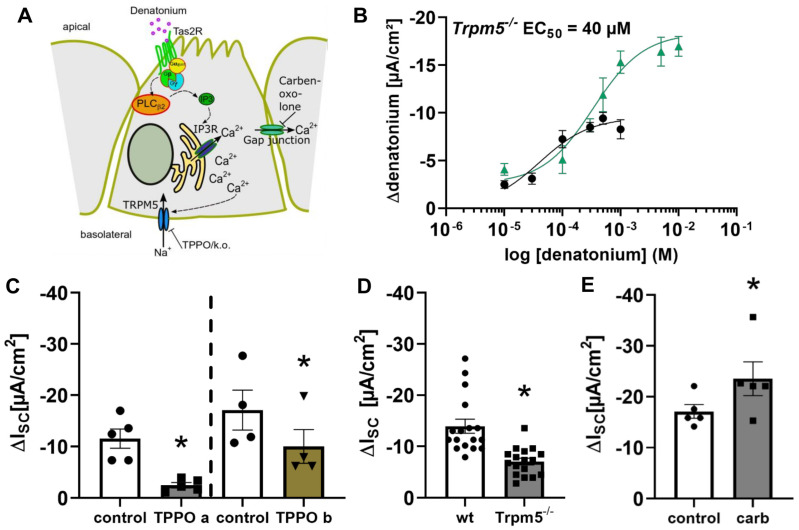
Role of the Trpm5 channel in the denatonium-induced current changes. (**A**) Schematic drawing of the bitter taste signalling cascade activated by denatonium in murine tracheal brush cells. (**B**) In Trpm5^−/−^ mice, the response to denatonium was concentration-dependent with an EC_50_ of 40 µM (*n* = 5 for each concentration, black curve). The corresponding curve from wildtype mice is depicted in green (data from Figure 1B). (**C**) The denatonium-induced current was reduced in the presence of the Trpm5 antagonist TPPO (triphenylphosphine oxide; 100 µM) when TPPO was applied apically or basolaterally in wildtype mice (∆I_SC_, *n* = 5, * *p* < 0.05). (**D**) Comparison of the apical application of denatonium (1 mM) in the tracheae of wildtype (wt) and Trpm5^−/−^ mice. The denatonium-induced effect (∆I_SC_) was reduced in Trpm5^−/−^ compared to wt mice (*n* = 17, * *p* < 0.05). (**E**) In the presence of the gap junction antagonist carbenoxolone (carb, 100 µM, apical and basolateral), the denatonium-induced effect was increased (ΔI_SC_, *n* = 5, * *p* < 0.05).

The involvement of the Trpm5 ion channel in the denatonium-induced current changes (Figure 5A) was addressed in tracheae from Trpm5^−/−^ mice by Ussing chamber experiments. The denatonium-induced current changes in Trpm5^−/−^ mice were concentration-dependent with an EC_50_ of 40 µM (Figure 5B, black curve), which can be explained by the fact that the maximum response in Trpm5^−/−^ mice was already reached at a concentration of 100 µM (Figure 5B, black curve), whereas the maximum response in wt mice was reached at 1 mM (Figure 5B, green curve). In line with this, we then investigated the denatonium-induced current changes in the presence of the Trpm5 inhibitor triphenylphosphine oxide (TPPO, 100 µM) in wt mice. Apical as well as basolateral application of TPPO led to a significant reduction in the denatonium-induced effect (Figure 5C). TPPO is highly lipophilic and therefore membrane permeable [35]. The stronger inhibition that was observed when TPPO was applied apically is most probably due to a better diffusion through the apical epithelial membranes compared to a basolateral diffusion through all layers of the tracheal wall. In line, Ussing chamber experiments revealed a reduced effect of denatonium (1 mM, apical) in Trpm5^−/−^ mice compared to wt mice (Figure 5D). Additionally, the gap junction inhibitor carbenoxolone (100 µM, apical and basolateral), which prevents the flux of Ca^2+^ and other messengers from one cell to another, increased the denatonium-induced effect (Figure 5E).

### 3.3. Activation of Brush Cells Leads to Cholinergic Paracrine Signalling

**Figure 6:**
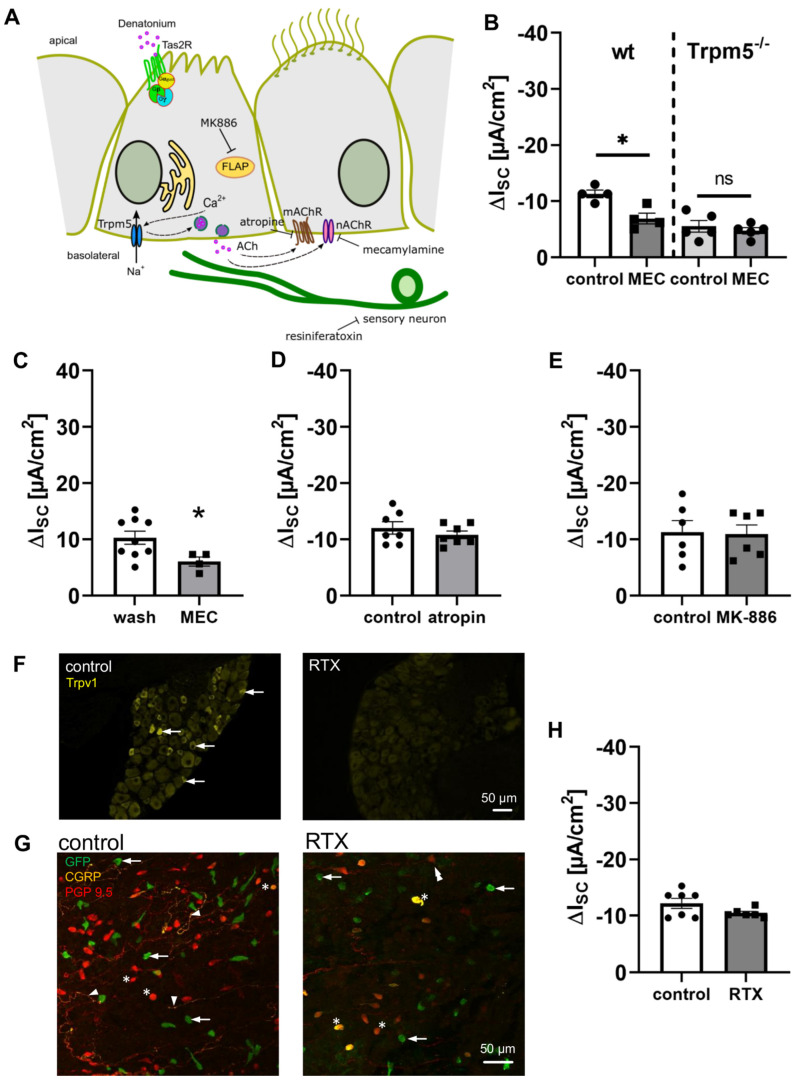
Cholinergic signalling is involved in the denatonium-induced effect. (**A**) Schematic drawing of the influence of the muscarinic ACh receptor inhibitor atropine, the nicotinic ACh receptor inhibitor mecamylamine, the FLAP antagonist MK-886, and the TRPV1 agonist resiniferatoxin. (**B**) In the presence of the nAChR antagonist mecamylamine (MEC, 25 µM, apical and basolateral), the denatonium-induced effect was significantly reduced in wildtype but not in Trpm5^−/−^ mice (∆I_SC_, *n* = 4–5, * *p* < 0.05, ns: not significant). (**C**) Application of MEC (25 µM, apical and basolateral) in the presence of denatonium (1 mM, apical) increased I_SC_, but to a lesser extent than the wash out of denatonium. (**D**) The mAChR antagonist atropine (50 µM, apical and basolateral) did not influence the denatonium-induced current (∆I_SC_, *n* = 5). (**E**) The FLAP inhibitor MK-886 (1 µM, apical and basolateral) did not alter the denatonium-induced effect (∆I_SC_, *n* = 6). (**F**) Immunostaining of DRG sections from an untreated and an RTX-treated wildtype mouse stained for Trpv1 (arrows: Trpv1^+^ neurons). (**G**) Immunostaining of a tracheal whole mount preparation of an untreated trachea from a Trpm5-tauGFP mouse and a trachea from a Trpm5-tauGFP mouse treated with resiniferatoxin (RTX) stained for brush cells (GFP, green, arrows) and CGRP (yellow) and the pan-neuronal marker PGP9.5 (red) (arrowheads: nerves, stars: neuroendocrine cells). (**H**) The denatonium-induced effect was not changed in RTX-treated mice compared to the control mice (∆I_SC_, *n* = 6–7).

We and others have recently shown that tracheal BC release ACh upon the activation of the bitter taste signalling cascade [9,10]. This in turn elicits paracrine effects on neighbouring cells, such as the modulation of the particle transport speed and ciliary beat as part of the mucociliary clearance [9,10]. To elucidate whether the observed effect of denatonium on the transepithelial ion transport was dependent on BC-mediated paracrine cholinergic signalling, we applied mecamylamine (25 µM, apical and basolateral), a nAChR antagonist, in order to inhibit cholinergic signalling after the release of ACh from BC (Figure 6A). In the presence of mecamylamine, the denatonium-induced effect was significantly reduced in wt but not in Trpm5^−/−^ mice (Figure 6B). Vice versa, the application of mecamylamine (25 µM, apical and basolateral) in the presence of denatonium increased I_SC_ to almost, but not completely, the same extent as wash out over the same time course (Figure 6C). In contrast to this, the application of the inhibitor atropine (50 µM, apical and basolateral), an antagonist of mAChR, had no influence on the denatonium-induced effect (Figure 6D). This indicates that the activation of BC leads to a release of ACh, which then acts in a paracrine manner on the ion and fluid transport in the trachea via an activation of nAChR on neighbouring cells. In contrast to this, mAChR are not involved in the paracrine BC-dependent cholinergic regulation of ion transport. 

It has recently been shown that the cholinergic chemosensory cells of the respiratory tract are able to synthetize cysteinyl leukotrienes [36]. Therefore, we addressed the hypothesis that the stimulation of BC with denatonium additionally leads to a release of cysteinyl leukotrienes. To prove this, we inhibited cysteinyl leukotriene synthesis with the 5-lipoxygenase-activating protein (FLAP) inhibitor MK-886 (1 µM, apical and basolateral) prior to denatonium application. Under these conditions, the denatonium-induced effect was not altered compared to the control conditions (Figure 6E).

Since BC are approached by cholinoceptive sensory nerve endings [14,37] and BC-released ACh induces the activation of nAChR on these nerve endings [10,14], we next investigated whether the denatonium-induced current changes were mediated by the stimulation of these nerve endings. Therefore, we ablated Trpv1^+^ sensory nerve fibres with resiniferatoxin and then performed Ussing chamber experiments with tracheae from these mice. Resiniferatoxin is a strong Trpv1 agonist, which leads to the ablation of Trpv1^+^ sensory nerve endings in mice when it is applied in high concentrations [38,39]. Immunostainings of DRG tissue sections for Trpv1 confirmed the successful ablation of sensory neurons after resiniferatoxin treatment, since we observed Trpv1^+^ neurons only in DRG of untreated control, but not in resiniferatoxin-treated mice (Figure 6F). The ablation of sensory nerve endings in the trachea was confirmed by the immunostaining of tracheal whole mount preparations for the neuropetide calcitonine gene-related peptide (CGRP) (Figure 6G). Indeed, we observed a dense nerve fibre network of CGRP^+^ and/or protein gene product (PGP) 9.5^+^ (a pan-neuronal marker) nerve fibres in untreated mice (left panel Figure 6G), which was not present after resiniferatoxin treatment. Few PGP9.5^+^ nerve fibres were observed in tracheae from resiniferatoxin-treated mice (right panel Figure 6G). In resiniferatoxin-treated mice, the denatonium-induced effect was not altered compared to the control mice (Figure 6H), indicating that substances released from sensory nerve endings via antidromic signalling are not involved in the denatonium-induced current changes.

### 3.4. Bitter Taste Signalling Leads to an Inhibition of cAMP Signalling Pathways

**Figure 7:**
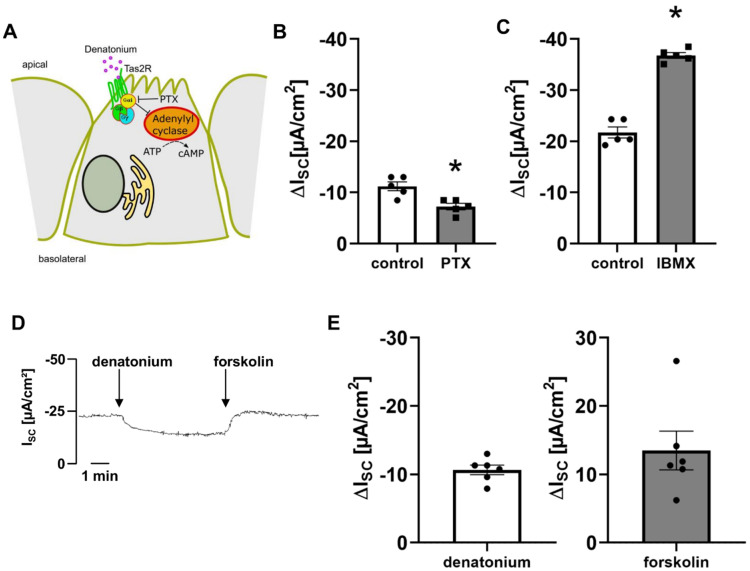
Denatonium influences cAMP-dependent ion currents. (**A**) Schematic drawing of the effect of pertussis toxin (PTX). (**B**) The inhibitor of the G_αi_ subunit pertussis toxin (500 ng/mL, apical) reduced the denatonium-induced current (∆I_SC_, *n* = 5, * *p* < 0.05). (**C**) In the presence of the phosphodiesterase inhibitor IBMX (100 µM apical), the denatonium-induced effect was increased (∆I_SC_, *n* = 5, * *p* < 0.05). (**D**) Apical application of 1 mM denatonium decreased I_SC_, which was reversed by forskolin (50 µM, apical). Representative current trace. (**E**) Forskolin increased I_SC_ in the presence of denatonium by approximately the same extent as it was decreased before with denatonium (∆I_SC_, *n* = 6).

The application of 500 ng/mL pertussis toxin (10 min prior to and during the incubation with denatonium), an inhibitor of G_αi_ as well as G_αgust_ subunits [19,40], decreased the denatonium-induced effect by 35.35% (Figure 7A,B). G_αi_ inhibits adenylyl cyclase, which catalyses the synthesis of cAMP from ATP [41], and G_αgust_ activates phosphodiesterases, decreasing cAMP levels [42]. The application of the phosphodiesterase inhibitor IBMX (100 µM, apical) increased the denatonium-induced effect (Figure 7C), while the application of the adenylyl-cyclase activator forskolin (10 µM, apical) restored the current to baseline conditions in the presence of denatonium (Figure 7D,E). Taken together, these data indicate that denatonium inhibits cAMP-dependent ion currents.

### 3.5. Bitter Taste Signalling Inhibits the Epithelial Sodium Channel

**Figure 8:**
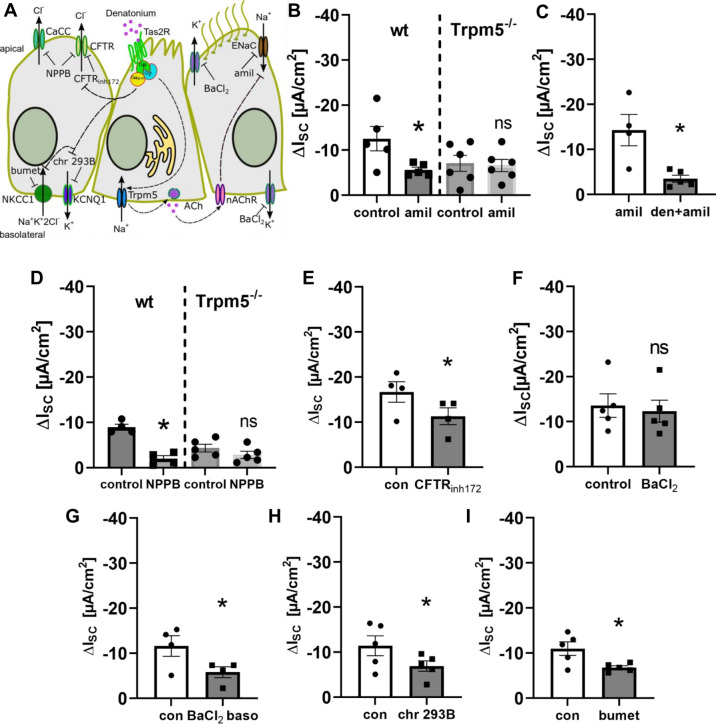
Involvement of ion channels in the denatonium-induced effect. (**A**) Schematic drawing of the influence of the epithelial sodium channel (ENaC) inhibitor amiloride, the Cl^−^-channel antagonist NPPB, the cystic fibrosis transmembrane conductance regulator (CFTR) blocker CFTR_inh172_, and the K^+^-channel inhibitor BaCl_2_ and the KCNQ1 channel antagonist chromanol 293B as well as the inhibitor of the Na-K-2Cl cotransporter 1 (NKCC1) bumetanide. (**B**) The ENaC inhibitor amiloride (10 µM, apical) reduced the denatonium-induced current in wildtype but not in Trpm5^−/−^ mice (ΔI_SC_, *n* = 5–6, * *p* < 0.05, ns: not significant). (**C**) The effect of amiloride (10 µM, apical) was significantly reduced in the presence of denatonium (1 mM apical) (ΔI_SC_, *n* = 4–5, * *p* < 0.05). (**D**) The Cl^−^-channel inhibitor NPPB (100 µM, apical) reduced the denatonium-induced effect in wildtype but not in Trpm5^−/−^ mice (ΔI_SC_, *n* = 5–6, * *p* < 0.05, ns: not significant). (**E**) The CFTR antagonist CFTR_inh172_ (10 µM, apical) reduced the denatonium-induced current (∆I_SC_, *n* = 4, * *p* < 0.05, con: control). (**F**) The non-selective K^+^-channel inhibitor BaCl_2_ (5 mM, apical) did not change the denatonium-induced current (∆I_SC_, *n* = 5, ns: not significant) compared to the control effect. (**G**) In the presence of 5 mM BaCl_2_ on the basolateral side of the epithelium, the denatonium-induced current was reduced (∆I_SC_, *n* = 4, * *p* < 0.05, con: control). (**H**) The KCNQ1 antagonist chromanol 293B (100 µM, basolateral, chr 293B) reduced the denatonium-induced current (∆I_SC_, *n* = 5, * *p* < 0.05, con: control). (**I**) The NKCC inhibitor bumetanide (200 µM, basolateral) reduced the denatonium-induced current (∆I_SC_, *n* = 5, * *p* < 0.05, con: control).

We next investigated which ion channels are modulated by BC activation (Figure 8A). In the presence of amiloride (10 µM, apical), an ENaC (epithelial sodium channel) inhibitor, the denatonium-induced effect was decreased by 55% in wt mice, but not in Trpm5^−/−^ mice (Figure 8B). Vice versa, the amiloride-effect was reduced in the presence of denatonium (Figure 8C). The reduced effects indicate that ENaC is indeed inhibited by the denatonium-induced signalling cascade in BC. 

Furthermore, the denatonium-induced current changes were significantly reduced in the presence of the chloride channel inhibitor NPPB (100 µM, apical) in wt but not in Trpm5^−/−^ mice (Figure 8D), indicating that denatonium inhibits apical chloride secretion. As our experiments with IBMX and forskolin point towards an involvement of cAMP in the denatonium-induced current changes, we next inhibited the CFTR chloride channel, which is regulated by cAMP, with the antagonist CFTR_inh172_ (10 µM, apical, Figure 8E). Indeed, we observed a reduction in the denatonium-induced current upon CFTR inhibition. In the presence of the general K^+^-channel inhibitor BaCl_2_ (5 mM, apical) the effects on denatonium-induced current changes were not altered (Figure 8F), indicating that apical K^+^-channels are not involved. In contrast to this, the denatonium-induced current changes were significantly reduced in the presence of BaCl_2_ (5 mM) on the basolateral side of the epithelium (Figure 8G). The application of BaCl_2_ itself lead to a decrease in I_SC_ of about 23%. In order to identify the basolateral K^+^ channel that is involved in the denatonium-induced current changes, we inhibited cAMP-dependent KCNQ1 channels with chromanol 293B (Figure 8H). In the presence of this antagonist, the denatonium-induced current changes were significantly reduced. Since apical chloride secretion via cAMP-dependent CFTR not only necessitates the activity of basolateral potassium channels, but also of the Na-K-2Cl cotransporter (NKCC) in the basolateral membrane [4], we next inhibited NKCC1 with bumetanide (200 µM, basolateral), which reduced the denatonium-induced current (Figure 8I). Taken together, these results indicate that denatonium inhibits the cAMP-activated ion channels CFTR and KCNQ1.

## 4. Discussion

**Figure 9:**
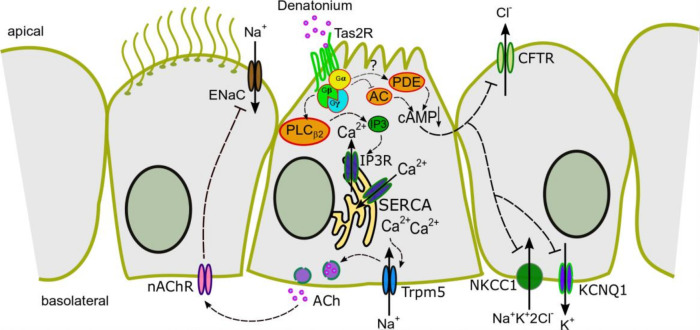
Proposed signalling pathway of the influence of denatonium on transepithelial ion transport. Denatonium binding to Tas2R in brush cells leads to a G_βγ_-dependent activation of PLC_β2_, which results in an activation of IP3 receptors. The increase in [Ca^2+^]_i_ leads to an activation of Trpm5 followed by a release of ACh. The released ACh then binds to nAChR on neighbouring cells, resulting in an inhibition of ENaC and chloride channels. Simultaneously, the G_α_-dependent reduction of [cAMP]_i_ in brush cells might lead to an additional decrease in ENaC, CFTR, and KCNQ1 as well as NKCC1 activity.

During the last decade, chemosensory cells have emerged as crucial regulators of innate immune responses [9,10,11,12,13,37,43]. Recently, we and others have shown that tracheal BC are able to regulate ciliary beating as part of the MCC [9,10]. To date, however, it has remained elusive whether BC are further involved in the regulation of transepithelial ion transport processes. In this paper, we clearly demonstrated that the activation of BC modulates the transepithelial ion transport-mediated component of MCC by ACh release (Figure 9). This signalling cascade involves the G_βγ_-dependent activation of PLC_β2_ and, subsequently, an IP3-dependent release of Ca^2+^ from intracellular stores. [Ca^2+^]_i_, which further activates Trpm5 ion channels triggering the release of ACh. ACh then acts in a paracrine manner on nAChR, ultimately inhibiting ENaC and apical chloride channels.

Recently, we demonstrated that the application of denatonium leads to an increase in [Ca^2+^]_i_ and, thus, is a suitable agonist for BC [10,14]. Now, we further clarified the involvement of the classical bitter taste signalling cascade in the denatonium-induced ion transport changes. The observed action via G_βγ_ signalling is consistent with the bitter taste signalling cascade in taste buds [44]. Tas2R108 is one of the most abundant proteins present in BC [10]. In line with Chandrashekar et al. [45], we confirmed that denatonium is a suitable agonist to Tas2R as shown by the increase in [Ca^2+^]_i_ in Tas2R108-expressing HEK293 cells. Generally, denatonium may act on a wide variety of Tas2R, e.g., the mouse taste receptors mTas2R105, mTas2R108 mTas2R123, mTas2R135, mTas2R140, and mTas2R144, as well as the human taste receptors hTAS2R4, hTAS2R8, hTAS2R10, hTAS2R13, hTAS2R39, hTAS2R43, hTAS2R46, and hTAS2R47 [45,46,47].

The increase in [Ca^2+^]_i_ evoked by Tas2R, G_βγ_, PLC_β2_, and IP3-dependent signalling consequently activates Trpm5, since this channel is Ca^2+^-sensitive [15,48]. Trpm5 has been established as a marker for BC due to its exclusive expression in this particular cell type in the airway epithelium [10,24,25,26,37,49]. Now, we demonstrated that Trpm5 is involved in the denatonium-induced ion transport changes. The maximum response was inhibited in Trpm5-deficient (Trpm5^−/−^) mice, although Trpm5^−/−^ mice revealed a lower EC_50_ in response to denatonium. The reduced maximum response in Trpm5^−/−^ mice emphasises the importance of a functional Trpm5 channel for fulfilling the BC-induced ion transport changes. Furthermore, the lower EC_50_ does not imply that Trpm5^−/−^ are more sensitive, but that the involved cAMP-dependent ion transport changes discussed below might play a role besides Trpm5-dependent pathways. This result is in line with observations by Damak et al. [20] who found that Trpm5^−/−^ mice responded to bitter compounds to a reduced extent when compared to wt mice, while the reaction was not completely abolished. The authors hypothesised that Trpm5-independent pathways might be mediated by other Ca^2+^-activated channels, by direct release of the neurotransmitter as a response to the increase in [Ca^2+^]_i_ or completely independent of Ca^2+^. Interestingly, while the regulation of the cilia-mediated part of mucociliary clearance by denatonium was found to be solely dependent on the Trpm5 ion channel [10], the regulation of transepithelial ion transport processes seems to be only partially mediated through Trpm5-dependent signalling, since the effect of denatonium was not completely abolished in Trpm5^−/−^ mice. Most sequencing studies did not find Tas2R as hallmarks for other airway epithelial cell types besides brush cells [24,25]. Additionally, we were able to detect only a weak expression of Tas2R in ciliated cells [10]. In taste buds, the activation of Trpm5 occurs via G_βγ_ signalling, which leads to an IP3-dependent Ca^2+^ secretion [44]. In addition to this, the G_α_ subunit decreases the intracellular cAMP concentration [42], which might lead to an inhibition in cAMP-dependent ion transport. Indeed, the denatonium-induced effect in our study was partly due to an inhibition of cAMP-dependent ion currents via G_α_ signalling as observed with PTX, IBMX, and forskolin. In addition to this, the regulation of ciliary beat frequency by bitter substances has previously been shown to be mediated by the ciliated cells themselves [50]. However, any role of chemosensory cells to this effect was not addressed in this study. An involvement of BC in the regulation of the cilia-mediated component of MCC in the lower airways was shown recently [9,10]. In the mouse trachea, denatonium activated the cilia-mediated part of MCC by increasing particle transport speed in a Trpm5-dependent manner [10]. We and others further demonstrated that bacterial formyl peptides as well as *Pseudomonas aeruginosa* quorum-sensing molecules increased ciliary beat in a BC-dependent manner [9,10].

To date, auto- and paracrine signalling of BC via ACh has been described [9,10] and it was suggested that IL25-release from BC plays a potential role as a signalling mediator of type 2 inflammation [24]. Additionally, cysteinyl leukotrienes are synthesised by BC [36]. Thus, the release of these mediators could also lead to paracrine ion transport changes. Here, we found that BC released ACh influences transepithelial ion transport processes via nAChR, but not mAChR and that cysteine leukotrienes are not involved. 

Our observation of a Tas2R-mediated change in the transepithelial ion transport in the mouse trachea is in line with a recent study employing sinonasal epithelial air liquid interface cultures [19]. This study described a G_α_ gustducin-mediated cAMP-dependent action on K2P channels [19]. Solitary chemosensory cells in the nose and BC in the trachea are both chemosensory cells, and yet, they display different characteristics and employ different functional mechanisms. Our experiments with pertussis toxin point towards a G_αi_-dependent inhibition of the adenylyl cyclase. Instead of the activation of K2P channels after stimulation with bitter substances, we observed an inhibition of ENaC and of chloride channels as discussed below.

ENaC is a key player to provide homeostasis of the airway surface liquid and plays an essential role also in other organs, such as the kidneys, e.g., in regulation of the blood pressure [51]. In general, the dysregulation of ENaC leads to severe symptoms in humans. While normal ENaC activity is essential for diverse physiological processes, inhibition of ENaC might be beneficial in pathologies with ENaC hyperactivation. The overactivity of ENaC in the kidney leads to Liddle’s syndrome, characterised by severe hypertension [51]. Additionally, in the airways, the overactivity of ENaC has deleterious effects on the airway surface liquid production. In cystic fibrosis, an inhibition of ENaC through CFTR is absent, which leads to a decrease in airway surface liquid height and to dehydration of the airways [52]. Interestingly, ENaC can be inhibited via cholinergic signalling through mAChR [53]. In this study, ENaC-dependent currents were partially inhibited by the cholinergic agonist carbachol in airway epithelial cells from different species. Here, we observed a reduction in ENaC current by denatonium. Joo and colleagues [53] observed that the carbachol-induced ENaC inhibition could be completely reverted by the inhibition of mAChR contrasting to our results that BC-evoked ion transport changes are mediated by nAChR and not by mAChR. Therefore, we propose that the ACh release from BC leads to an inhibition of ENaC via paracrine signalling. However, we cannot rule out that the additional inhibition of ENaC might occur via the observed decrease in cAMP-levels. Since cAMP is potentially spreading through gap junctions [54,55,56] and stimulating ENaC, the lower levels of cAMP might lead to its inhibition [57,58]. Thus, the activation of Tas2R in BC might represent a useful tool for regulating ENaC activity. Furthermore, since the expression of these receptors has also been shown in primary renal epithelial cells [59], it is tempting to speculate that bitter receptor agonists could also have beneficial effects in the kidneys as ENaC inhibitors to regulate blood pressure.

In addition to an ENaC modulation, our experiments with the chloride channel inhibitor NPPB point towards an inhibition of chloride channels. The simultaneous inhibition of chloride secretion and sodium absorption might be necessary to remain homeostasis of the airway surface liquid and could be beneficial to prevent excess fluid accumulation in the airways. A possible underlying mechanism could be the decreased level of cAMP after BC activation since the CFTR chloride channel is activated by cAMP. Indeed, our experiments with the CFTR inhibitor CFTR_inh172_, point towards an inhibition of CFTR by denatonium. Additionally, while the activation of apical α3β4 nAChR leads to an activation of TMEM16A [18], inhibiting effects on CFTR by α7 nAChR have been previously described in the airway epithelium [60]. Apical chloride secretion is often driven by basolateral potassium secretion in the airway epithelium and the cAMP-regulated potassium channel KCNQ1 is present on the basolateral side of the mouse tracheal epithelium [61]. Thus, it is likely that basolateral potassium secretion is inhibited simultaneously to apical chloride secretion by denatonium in order to maintain homeostasis. Indeed, our experiments with the KCNQ1 inhibitor chromanol 293B indicate an inhibition of this channel mediated by denatonium. Additionally, the NKCC1 cotransporter, located in the basolateral membrane of the tracheal epithelium, plays an important role in the regulation of apical chloride secretion and especially of cAMP-dependent chloride secretion via CFTR [4]. Our observations of a reduced denatonium-induced current upon NKCC1-inhibition, suggest that the inhibition of NKCC1 by denatonium is likely induced by decreased cAMP levels. Therefore, we conclude that the reduction in cAMP after the activation of BC with denatonium leads to an inhibition of the cAMP-dependent ion channels CFTR and KCNQ1, and the NKCC1 co-transporter. 

In conclusion, we described a novel mode of action by tracheal BC to release ACh upon stimulation with Tas2R agonists, which ultimately resulted in decreased sodium reabsorption via ENaC accompanied by decreased chloride secretion apically and the simultaneous inhibition of basolateral potassium secretion. Thus, the pharmacological targeting of BC could be a useful strategy in contributing to the homeostasis of airway surface liquid.

## Data Availability

The data generated in this study are available from the corresponding author upon reasonable request.

## Supplementary Materials

The following supporting information can be downloaded at: www.mdpi.com/article/10.3390/cells11152411/s1, Table S1 Primer sequences.
