# Supplementary material for: Taste Receptor Activation in Tracheal Brush Cells by Denatonium Modulates ENaC Channels via Ca2+, cAMP and ACh
Source: Cells. 2022 Aug 4;11(15):2411. doi: 10.3390/cells11152411 (PMC9367940; doi:10.3390/cells11152411)
Supplement: Supplementary file 1 [file cells-11-02411-s001.zip › cells-1806634-supplementary.pdf]

Supplementary Table S1: Primer sequences

MX18748	CATGGTGGCTAAACGCTAGCCAGCTTGGGTCTC
MX18749	TAGCCGCTGATCAGCCTCGACTGTGCC
MX18750	GCTAGCGTTTAGCCACCATGCTGAGTGCGGCAGAAGGCATC
MX18751	TCGAGGCTGATCAGCGGCTAAAAGAACTTTAATCCTTGCAGTAC
MX18752	GCTAGCGTTTAGCCACCATGCTCTGGGAACTGTATGTATTTG
MX18753	TCGAGGCTGATCAGCGGCTACTTGTAGAAACAGAAAATCTTC
